# Participant engagement and involvement in longitudinal cohort studies: qualitative insights from a selection of pregnancy and birth, twin, and family-based population cohort studies

**DOI:** 10.1186/s12874-024-02419-8

**Published:** 2024-12-03

**Authors:** Isabelle Budin-Ljøsne, Nanna A. G. Fredheim, Charlotte Alison Jevne, Bojana Milanovic Kleven, Marie Aline Charles, Janine F. Felix, Robin Flaig, María Paz García, Alexandra Havdahl, Shahid Islam, Shona M. Kerr, Inger Kristine Meder, Lynn Molloy, Susan M. B. Morton, Costanza Pizzi, Aamnah Rahman, Gonneke Willemsen, Diane Wood, Jennifer R. Harris

**Affiliations:** 1 Department of Food Safety, Norwegian Institute of Public Health, P.O. Box 222, Skøyen, Oslo, NO-0213 Norway; 2 Kristiania University College, Oslo, Norway; 3 Department of Health Data Reception, Norwegian Institute of Public Health, Oslo, Norway; 4 Research Administrative Support, Norwegian Institute of Public Health, Oslo, Norway; 5 Ined Inserm EFS Joint Unit Elfe, Aubervilliers, France; 6 The Generation R Study Group, Erasmus MC, University Medical Center Rotterdam, Rotterdam, The Netherlands; 7 Department of Pediatrics, Erasmus MC, University Medical Center Rotterdam, Rotterdam, The Netherlands; 8 Usher Institute, University of Edinburgh, Edinburgh, UK; 9 Department of Twin Research & Genetic Epidemiology, King’s College London, London, UK; 10 PsychGen Centre for Genetic Epidemiology and Mental Health, Norwegian Institute of Public Health, Oslo, Norway; 11 Nic Waals Institute, Lovisenberg Diaconal Hospital, Oslo, Norway; 12 Department of Psychology, PROMENTA Research Centre, University of Oslo, Oslo, Norway; 13 Bradford Institute for Health Research, Bradford Royal Infirmary, Bradford, UK; 14 Medical Research Council Human Genetics Unit, University of Edinburgh, Edinburgh, UK; 15 Statens Serum Institut, Danish National Biobank, Statens, Denmark; 16 Population Health Sciences, Bristol Medical School, ALSPAC (Children of the 90s), University of Bristol, Bristol, UK; 17 Research Institute for Innovative Solutions for Well-Being and Health (INSIGHT), Faculty of Health, University of Technology, Sydney, Australia; 18 Department of Medical Sciences, University of Turin, Turin, Italy; 19 Bradford Teaching Hospitals NHS Foundation Trust, Bradford, UK; 20 Department of Biological Psychology, Vrije Universiteit Amsterdam, Amsterdam, The Netherlands; 21 The Raine Study, School of Population and Global Health, The University of Western Australia, Perth, Australia; 22 Centre for Fertility and Health, Norwegian Institute of Public Health, Oslo, Norway

**Keywords:** Cohort studies, Twin(s), Participant engagement and involvement, Longitudinal studies

## Abstract

**Background:**

Longitudinal cohort studies are pivotal to understand how socioeconomic, environmental, biological, and lifestyle factors influence health and disease. The added value of cohort studies increases as they accumulate life course data and expand across generations. Ensuring that participants stay motivated to contribute over decades of follow-up is, however, challenging. Participant engagement and involvement (PEI) aims to secure the long-term commitment of participants and promote researcher-participant interaction. This study explored PEI practices in a selection of pregnancy and birth, twin, and family-based population cohort studies.

**Methods:**

Purposive sampling was used to identify cohorts in Europe, Australia and New Zealand. Fourteen semi-structured digital interviews were conducted with cohort study representatives to explore strategies for participant recruitment, informed consent, communication of general and individual information to participants, data collection, and participant involvement. Experiences, resources allocated to PEI, and reflections on future PEI, were discussed. The interview data were analyzed using a content analysis approach and summary results were reviewed and discussed by the representatives.

**Results:**

The cohort studies used various strategies to recruit participants including support from health professionals and organizations combined with information on the studies’ web sites and social media. New approaches such as intra-cohort recruitment, were being investigated. Most cohorts transitioned from paper-based to digital solutions to collect the participants’ consent and data. While digital solutions increased efficiency, they also brought new challenges. The studies experimented with the use of participant advisory panels and focus groups to involve participants in making decisions, although their success varied across age and socio-economic background. Most representatives reported PEI resources to be limited and called for more human, technical, educational and financial resources to maximize the positive effects of PEI.

**Conclusions:**

This study of PEI among well-established cohort studies underscores the importance of PEI for project sustainability and highlights key factors to consider in developing PEI. Our analysis shows that knowledge gaps exist regarding which approaches have highest impact on retention rates and are best suited for different participant groups. Research is needed to support the development of best practices for PEI as well as knowledge exchange between cohorts through network building.

**Supplementary Information:**

The online version contains supplementary material available at 10.1186/s12874-024-02419-8.

## Background

Many countries have established large, longitudinal cohort studies that follow research participants sharing common characteristics over many years to understand how genetic, biological, socioeconomic, environmental and lifestyle factors affect disease risk and well-being [1–4]. Longitudinal cohort studies take various forms and include diverse groups of population. Pregnancy and birth cohort studies follow children born in specific time periods, as well as mothers and in some cases, partners [5]. Twin studies investigate similarities and differences between identical and fraternal twins over time [6] whereas family-based population cohort studies may focus on groups of population living in a specific area or sharing common ancestry. Longitudinal cohort studies provide countless groundbreaking insights into disease development, gene-environment interactions and well-being across the life course [7, 8]. Creating synergies across these resources has led to various initiatives targeted towards building complementarity and interoperability of data, and for developing national, pan-European and broader international infrastructures to leverage the data in science [9, 10]. Longitudinal cohort studies provide an essential evidence-base for informing health policy development and played a critical role during the Covid-19 pandemic. The extensive investments made by researchers and funders to establish and maintain longitudinal cohort studies continue today and their added value will increase as the participants transition though their life-course and new health problems emerge, providing an ever-richer set of longitudinal information [11]. This entails the extension across time and, for family studies, expansion across generations of children, parents, and grandparents.

Given the major role longitudinal cohort studies play in modern epidemiological and life-course research, securing the long-term commitment of the participants over time is critical. Longitudinal cohort studies are living entities and they must interact and engage with their participants to ensure long-term sustainability. Cohort studies have reported attrition rates varying between 30 and 70% over time [12]. High attrition rates jeopardize the reliability of any study as the remaining sample becomes smaller and less representative of the cohort, thus leading to risks of bias [13]. Recruiting and retaining participants in longitudinal cohort studies is however challenging. Participation requires committing over an extended period, and participants may lose interest, experience that their changing living circumstances render participation difficult, or feel that they receive too many competing demands to participate in such studies [14]. Recruiting participants with various ethnic and socio-economic background can be particularly demanding if these groups do not trust public health authorities or are approached too often [15]. Participant engagement and involvement (PEI) aims to encourage and secure the long-term commitment of participants and promote trustworthy researcher-participant interaction [16, 17]. Recent systematic reviews have highlighted the importance of tailoring PEI to the specificities and needs of different participant groups and reducing participant burden [18–20], and studies have explored barriers and facilitators to PEI [21] among various participant groups [22], including young people [23, 24], ethnic minorities [25] and older participants [26, 27]. However, relatively little practical guidance exists regarding how to develop approaches in longitudinal cohort studies relevant to the specific contexts of the participants. Guidelines for PEI exist but remain largely generic [28–30].

Traditionally, longitudinal cohort studies have focused on *engaging* participants by routinely informing them about study progress and outcomes, for instance, through the study’s website, newsletter [23] and social media. The consent process can also be seen as a component of participant engagement as it aims to introduce participants with the study, familiarize them with what participation entails and describe potential risks and benefits of participation. Participant engagement is important to ensure good communication between researchers and participants, minimize attrition and build trust [22]. More recently, cohort studies have started to *involve* participants in the design and plans of the study. Participant involvement requires a more proactive role of cohort participants, who can influence the way the research develops by providing their insights and experiences, for instance, as members of advisory panels or steering groups [16] or in co-creation activities [31]. Ideally, one could develop participant engagement and involvement (PEI) strategies that involve several aspects of the study procedures, providing participants greater flexibility to manage consents, access information from devices and wearables, obtain feedback, and interact with researchers. Although PEI is context-dependent, there are many commonalities across longitudinal cohort studies regarding their reasons for developing PEI, and the nature of the studies and their participant base. Mapping the various approaches used to engage and involve participants and examining their experiences may bring useful new knowledge. In this paper, we investigate the types or PEI strategies used by a selection of large birth cohort studies, twin cohort studies and family-based population cohort studies. We focus on main activities conducted throughout the lifecycle of a study spanning participant recruitment, consent, data collection, communication and interaction strategies. We describe the types of resources allocated to such work, needs and aspirations for future PEI, and suggest potential avenues for the development of best practices for PEI.

## Methods

### Sample selection

During autumn 2020, our core research team (IBL, NGF, CJ, BMK) consulted the websites of the EU Child Cohort Network [10], the CHICOS network [32], the BIRTHCOHORTS network [33], and the Cohort and Longitudinal Studies Enhancement Resources (CLOSER) [34] to identify relevant birth cohort studies, twin cohort studies and family-based population cohort studies to invite. A purposive sampling approach was adopted to identify cohort studies. The core research team selected cohorts to invite based on the following criteria: the cohort studies featured PEI strategies on their web sites (e.g., participant panels, participant workshops, or participant portal), the cohorts included participants of different ages and generations and/or included diverse groups of participants in terms of ethnicity and cultural backgrounds. Fifteen cohorts in Europe, Australia and New Zealand gathering participants from different age groups were selected using this approach.

### Data collection and analysis

We sent an e-mail to the cohorts’ principal investigators with an invitation to participate in the study and requested that they appoint at least one staff representative who was knowledgeable about, or in charge of, PEI. Between March and August 2021, the core research team (IBL, NGF, CJ, BMK) conducted digital semi-structured interviews in English, using Microsoft Teams video calls, with the representatives for each study separately. The interviews lasted on average one hour. An interview guide was developed by the core research team, based on a review of the literature and discussions regarding the most important aspects to explore. The interview guide included 11 core questions to investigate methods used by the cohort studies to recruit participants and collect their consent, provide them with general and individual study results, collect their data and involve them in the life of the study. Additional questions asked about experiences of using PEI methods, including barriers and limitations, resources dedicated to PEI and needs for future PEI (Supplementary material). The interview guide was shared with the study representatives before the interview took place to give them enough time to prepare and consult colleagues. The study protocol was reviewed by the Data Protection Officer of the Norwegian Institute of Public Health and assessed not to require approval by an ethics committee. The representatives provided their written informed consent.

The interviews were audio recorded and transcribed verbatim by the three first authors (IBL, NGF, CJ). The interview data were analysed using a simple content analysis approach. For each interview, we reviewed the transcript and extracted the relevant content, which was summarized and categorized following the structure of the interview guide questions. The content was then fed into summary reports, one per cohort study, and the reports were sent to the respective representatives who reviewed the content of the reports and provided any missing information. An Excel table listing the PEI strategies mentioned across interviews was also shared with the representatives. Each representative was asked to tick-off which strategies had been used by his/her cohort to recruit participants, collect their informed consent and data, to provide participants with information and to involve them in the life of the study. In December 2021, the data from the summary reports were pooled in a matrix with the questions from the interview guide listed in rows and the summary information from the summary reports approved by the representatives listed in columns. In December 2021, the research team (IBL, NGF, CJ) gathered at a face-to-face workshop in Oslo to review the matrix content and produced a summary of results across cohorts. In April 2022, a digital workshop was organized to review and discuss the summary with the representatives to ensure that it correctly captured all information elements. The representatives were also invited to contribute to the writing of this paper.

## Results

We invited 15 cohort studies to participate and 14 of these (93%) agreed. The cohorts in our sample include 10 pregnancy and birth cohort studies, two twin cohort studies and two family-based population cohort studies. The main characteristics of the cohorts are described in Table 1. The pregnancy and birth cohort studies recruited their original participants between 1989 and 2021. Three of these cohorts are currently recruiting new participants, either across generations or among new groups of population. The two twin cohorts started original recruitment in the late eighties and are still recruiting new participants. Of the two family-based population studies, one is recruiting new groups of participants while the other recently completed recruitment.

**Table 1:** Main characteristics of cohorts

Cohorts	Country	Outreach	Original recruitment	Recruitment after original recruitment	Estimated total size per 01.01.2022	
**Pregnancy and birth cohort studies**						
Avon Longitudinal Study of Parents and Children (ALSPAC) [64, 65]	United Kingdom	Local	14,500 pregnant women recruited in 1991–1992	Recruited 3 generations over time: G0: Original parents (> 60 years of age) G1: Second generation (> 30 years of age) G2: Third generation (Children of the 90s participants) Currently recruiting G2 babies and children.	20,000	
Born in Bradford (BiB) [66, 67]	United Kingdom	Local	12,400 pregnant women and 3,400 partners recruited between 2007 and 2011	Currently recruiting pregnant women to: -The Born in Bradford Better Start (BIBBS) cohort study - The BIB4ALL data linkage cohort study.	30,000	
Danish National Birth Cohort (DNBC) [68]	Denmark	National	100,000 pregnant women recruited between 1996 and 2002	Planning to recruit next generation of pregnant women and their partners. In May 2019, half of the young participants in the DNBC had turned 20 years old.	180,000	
The French national cohort of children (ELFE) [69]	France	National	18,000 children born in 2011 recruited	No. The children in the original cohort are 13 years old. Their mothers are aged 28–59 years.	21,200	
Generation R [70]	The Netherlands	Local	9,778 pregnant women recruited between 2002 and 2006	Between 2017 to 2021, recruited 3,600 pre-conceptional women, early pregnant women, and partners to the Generation R Next cohort study.	18,000 in Generation R, 8,000 in Generation R Next	
Geração 21 [71]	Portugal	Local	8,600 newborns and their families recruited between 2005 and 2006	No. The children are now approx. 20 years old.	Not reported	
Growing up in New Zealand [72, 73]	New Zealand	National	6,800 newborns recruited between 2009 and 2010	No. The children are now approx. 14 years old.	18,000	
The Norwegian Mother, Father and Child Cohort Study [74]	Norway	National	112,500 pregnant women and partners recruited between 1999 and 2008	No. The children are now between 15 and 25 years old.	284,276	
Nascita e INFanzia: gli Effetti dell’Ambiente (NINFEA) [40]	Italy	National	7,500 pregnant women recruited between 2005 and 2016	No. The children are now between 8 and 19 years old.	14,300	
The RAINE study [75, 76]	Australia	Regional	2,900 pregnant women recruited between 1989 and 1991	Recruited 3 generations over time: GO: Grandmothers of the original children G1: Pregnant women G2: Children (between 33 and 35 years old) G3: Children of the children Currently recruiting the children (G3) of the children born into the study (G2) and their partners. Mothers currently aged 33 to 35 years.	7,000	
**Twin cohort studies**						
The Netherlands Twin Register [77]	Netherlands	National	120,000 minor and adult twins and equivalent number of relatives recruited since 1987	Currently recruiting newborn twins and multiples and their parents to the Young NTR cohort study.	280,000	
Twins UK [78]	United Kingdom	National	15,000 twins recruited since 1992	Ongoing recruitment of new twins. Mean age of cohort is 59 years.	15,000	
**Family-based population cohort studies**						
Generation Scotland [79]	United Kingdom	National	24,000 adults from approx. 7,000 families recruited since 2006	Currently recruiting new families and family members aged 12 and older who live in Scotland.	24,000	
Viking Genes Cohorts [80, 81]	United Kingdom	Regional	10,000 individuals aged 16 or older recruited since 2005 across 3 cohorts	No. The participants are now aged 16 to 101 years.	10,000	

The cohort representatives we talked to included PEI managers, project managers and/or principal investigators of the studies who were knowledgeable about the PEI activities conducted. Main PEI strategies employed by the studies are summarized in Table 2 and described below.

**Table 2:** Main PEI strategies used by the cohorts

Main participant engagement and involvement strategies	All cohorts (*N* = 14)
**Strategies to recruit participants**	
*Targeting any potential participants*	
Information via health care services/ maternity units	11
Information in media and press, including billboards	9
Information on cohort’s website, social media, newsletter	8
Information via local organizations, charities and communities	7
Direct contact with participants (e.g., letter, phone call, home visits)	7
Information via cohort participants and family members	7
*Targeting underrepresented groups*	
Information disseminated in specific areas (e.g., rural communities)	5
Information provided in community languages	4
Physical presence in local communities (e.g., mosques, gym for women)	4
**Strategies to collect the participants’ informed consent**	
Paper-based consent	9
Electronic consent	8
Consent renewal in connection with data collection waves	8
Reconsent of minor participants at 16 or 18 years of age	7
Information about possibility to opt-out at age of majority (18 yoa)	2
**Platforms to provide information to participants**	
*General information*	
Cohort’s website	14
Electronic or paper-based newsletter	14
Emails to participants	13
Workshops / public meetings	12
Videos / podcasts	11
Social media (Facebook, Instagram, Twitter)	11
Birthday cards / Greeting cards	8
*Individual information (e.g.*, *results from measurements)*	10
**Strategies to collect participants’ data**	
Online questionnaires	13
Data linkages	13
In-person measurements	11
Paper-based questionnaires	10
Mobile devices / wearables	8
Postal samples	8
Application on mobile phone	4
Use of reminders (e.g., by email, text message, phone call, home visit)	12
Use of incentives and/or thank-you gifts, lotteries	10
**Strategies to involve participants in the studies’ life**	
Participant advisory panels	10
Focus groups	8
Participant ambassadors	6
Use of incentives and/or thank-you gifts, lotteries	7

### Strategies to recruit participants

The cohort studies used a variety of methods to recruit their original participants. The pregnancy and cohort studies primarily recruited participants with help from medical professionals (e.g., midwives, general practitioners) and health care services (e.g., hospital wards, maternity units, mental health services). Local organizations, charities and communities (e.g., mother groups, pregnancy forums, parent organizations, baby congratulation services, school networks) were also used to spread information. One twin cohort study recruited participants through city council offices whereas the other one primarily recruited participants through media campaigns. Of the two family-based population studies, one allied with general practitioners to recruit the original cohort participants and the other one used a combination of press releases, public talks, and articles in regional newspapers to attract potential participants. Most cohorts also experimented the use of information on websites, social media, newsletters, newspaper articles, billboards, and posters. The representatives experienced that establishing alliances with other actors such as health care professionals and local organizations to recruit participants, was very effective. They found it more difficult to evaluate the impact of using social media and information in the press, although temporary peaks in recruitment could be observed following the use of such strategies.

A few cohort studies (*N* = 5) aimed to recruit participants from underrepresented populations, e.g., various ethnic groups. These studies produced information in different languages, which was shared at ethnic community events, in minority press, in neighborhoods of low socio-economic status, mosques, or gyms for women. The representatives however could not tell whether such strategies had a real impact on recruitment. They experienced that ethnic minority groups could be reluctant to participate in research because of past abuse in research settings and lack of trust in public authorities. Other representatives explained that their cohorts gathered homogeneous groups of participants, usually white Caucasians, and should aim at reaching new groups in the future. The representatives were however unsure of which methods would be best suited to do so.

Several pregnancy and birth cohort studies in our sample aimed to become multi-generational and recruit the children of the children with help from already enrolled participants. For instance, they asked mothers to recruit their grandchildren, together with partners. The representatives however explained that the use of such recruitment channel was new to them, and they did not have sufficient data yet to assess the effectiveness of such approach.

### Strategies to collect the participants’ informed consent

All cohorts collected the written informed consent of their participants. Practices for seeking the assent or consent of children largely varied between cohorts and were guided by applicable national legal requirements. A few pregnancy and birth cohort studies started collecting child assent from the age of 8 or 9 years, whereas most cohorts started doing so when the children were approximately 12–13 years old. When the child’s assent was sought, parental consent was also required. In most cohort countries, the children could provide their individual consent to participation from the age of 16 years and in such case, parental consent was not needed anymore. The representatives saw as important to collect the assent/consent from children as early as possible to give them the opportunity to express own willingness to participate in the study, and to create awareness of such participation. Several pregnancy and birth cohort studies had routines to seek the renewed informed consent of children once they became adults, except for two studies which only informed the children who had reached 18 years of age that they participated in the cohort and had the possibility to opt-out.

The cohort studies collected the participants’ informed consent or assent using paper-based (*N* = 9) and/or digital solutions (*N* = 8). Some studies recently transitioned to the use of digital consent in connection with Covid-19 surveys, which could only be conducted online due to country lockdowns. To collect digital consent from participants, several cohort studies used platforms provided by external providers. One cohort study developed its own digital platform and another integrated the consent process in electronic questionnaires to participants. Most representatives supported such digital transition as they believed it could potentially reduce the administrative and time burden, enable cost savings, and increase data security. Concerns were however raised that digital consent may not be accessible to participants without internet connection or technical skills.

Half of the cohort studies (*N* = 7) routinely collected the renewed consent of their participants for each new wave of data collection. Their representatives saw this as an opportunity to update contact details, broaden the terms of the consent, or maintain clarity about participation. In all cohort studies, the participants were informed about the right to withdraw and procedures for withdrawal via the study’s website. Some studies offered participants the possibility to skip a data collection wave or, in case of withdrawal, invited participants to an exit meeting, usually with a phone call, to inquire about reasons for withdrawal. The representatives reported that, in most cases, withdrawal was due to the participants’ lack of time or loss of interest.

### Strategies to provide general information to participants

All cohort studies had a study website to inform participants about research activities and results. The representatives believed having a website was useful to reach all participants, in particular the older ones. Several birth and cohort studies differentiated the content of their website between generations by providing pages targeting, for instance, parents or children. The representatives believed this brought added value to the website. All cohort studies produced newsletters at a frequency varying between one to four times a year. Most cohorts sent the newsletters to their participants using emails or published the newsletters on their website. A few cohorts also sent hard copies of the newsletters in the mail to their participants. The representatives believed the newsletters to be quite popular. It was however mentioned that keeping an updated registry of addresses (electronic or post) where to send the newsletters could be challenging as people move a lot or change email addresses. One representative explained that the cohort study organized small competitions to encourage participants to update their contact details.

The cohort studies sent emails to their participants, either to share newsletters and questionnaires, or to give specific updates. The representatives explained that it was difficult to know whether the emails were read by participants, were blocked by spam filters, or were sent to addresses that were not valid anymore. Several representatives worried that sending too many emails may lead to fatigue among participants, and possibly also contribute to the participants leaving the cohort study. One representative also experienced that young participants do not read emails.

Although most cohort studies used social media to connect with different audiences and age groups, four studies did not use them to protect the privacy and anonymity of the participants. The representatives believed that using Facebook likely had most impact to engage participants, disseminate research, and inform about recruitment waves. The representatives from the pregnancy and birth cohort studies suspected that Facebook might be more efficient to reach the parents’ generation and less useful to reach the younger participants, although they had not systematically collected data to document this. The representatives however experienced the use of social media as time consuming as frequent updates are needed, and demanding as posting information on such platforms requires expertise. They also found it difficult to know which social media are preferred by which groups of participants and which age groups.

Other communication methods included public lectures, podcasts, blogs, cohort anniversaries, digital photo exhibitions, community events such as workshops, face-to-face or digital conferences, play groups for mothers and children that the cohort researchers attend, and greeting cards in connection with birthdays or season holidays. The representatives reported that it was challenging to strike the right balance between offering activities and providing participants with sufficient and relevant information, and not overloading participants.

### Strategies to provide individual results to participants

Most cohort studies provided individual results to participants such as results from pregnancy ultrasound, blood testing, heart measurement and body composition scan, either directly by letter, email or via login to a national health portal. In some cohort studies, medical results could also be sent to the participants’ medical doctor, or directly to participants who were advised to consult their doctor. Several studies had established policies for the handling of incidental findings, for instance in connection with scans, and one study also provided individual genetic research results of clinical significance to participants [35]. Largely, the representatives experienced that providing individual results was appreciated by participants. One representative experienced that providing such results contributed to help individuals who suffered from severe conditions but were not aware of it. The representatives also believed that providing individual results contributed to the participants feeling rewarded for their attendance. Several representatives mentioned that providing individual results was an impactful incentive when recruiting participants and was an opportunity for the cohort studies to establish useful collaborations with health care professionals. The representative however experienced that providing individual results to participants was resource demanding and sometimes difficult because researchers were not trained to communicate this information to individual participants and clinicians.

### Strategies to collect participant data

The cohort studies primarily used electronic (*N* = 13) and paper-based questionnaires (*N* = 10), data linkages (*N* = 13) and/or in-person measurements (*N* = 11) to collect data from participants. The representatives explained that the use of electronic questionnaires was boosted by the Covid-19 pandemic and was seen as necessary to save costs and increase efficiency and data quality. Several representatives however worried that online data collection may exclude participants with low digital literacy or without internet access.

The cohorts collected data from medical health records, administrative datasets, health insurance databases and in a few cases, social media and transactional data (*N* = 13). The representatives experienced that data linkages could be challenging due to evolving legal requirements following the implementation of the European General Data Protection Regulation. One representative explained that it at times was unclear whether the scope of the informed consent was broad enough to cover linkages to other types of datasets. Consent to data linkages could also be interpreted differently by data controllers, which may stop linkages.

In-person measurements at clinics and research centres or in connection with home visits were still seen as important by the representatives to create contacts and relationships with the participants. Several cohort studies were gradually testing the use of postal kits sent to participants to collect biological samples, e.g., saliva. One representative explained that such method is efficient but requires that the participants understand how to use the kits and do not make any mistake when manipulating them. Other cohorts (*N* = 8) invited participants to wear devices that collect data automatically. The representatives believed that the devices were useful but that their use can be demanding. For instance, data interpretation can be difficult, the devices may not be worn by the participants if not attractive enough, and equipping participants may be costly and requires staff and logistical effort.

Only a few cohort studies (*N* = 4) used applications downloaded on the participants’ mobile phone to collect lifestyle data. One representative explained that developing the apps had been a huge undertaking due to stringent legal, safety and technical requirements. Other representatives were hesitant to develop apps as they feared that people may quickly loose interest in them. They also questioned the representativeness of data collected through apps if not all participants have a smart phone.

To help increase response rates, most cohorts (*N* = 12) used reminder emails and text messages, usually sent within a 1–2-week time span after the initial invite, or via phone calls and home visits. The representatives believed that reminders work well but should be used with caution not to create frustration among participants. Most cohort studies (*N* = 10) routinely provided participants with financial incentives. These included hourly-based payment, thank-you gifts of limited financial value, lotteries, or compensation for time and travel expenses. One study offered young participants free courses to prepare for theoretical driving exams. The representatives however found it difficult to measure the impact of employing such practices and, in the absence of data to document such impact, struggled to convince funders to support these practices.

### Strategies to involve participants in the studies’ life

Most cohort studies (*N* = 10) had established participant advisory panels to involve participants in the life of the study. The composition of the panels largely varied between cohorts and could include groups of parents with children, young participants alone, or participants from a defined geographical area. The size of the panels also varied from 10 to 20 to up to 200 participants. The panel participants were usually involved over several months or years and met regularly to discuss topics such as strategies for data collection, participant-researcher interaction, and the design of research activities. A few cohorts also gathered ad-hoc focus groups with adolescents or parents depending on project needs. Some studies (*N* = 5) appointed participants to become study ambassadors and represent their peers in the media, or asked participants to tell their stories on the study’s website.

The representatives strongly believed that involving participants was useful to develop new ideas and create a sense of belonging among participants. One representative explained that the participants often raised issues not thought of by the researchers and thus brought important perspectives. Another representative explained that during the Covid-19 pandemic, participants in the cohort panel played a crucial role in promoting the cohort’s surveys within their communities. Concerns were however raised that participants engaged in panels or focus groups as well as participant ambassadors were not representative of the full cohort as they often were highly educated young women of European ancestry. Engaging participants with different profiles was experienced as difficult. One representative also explained that establishing a group of engaged participants requires a lot of time to create trust and ensure that the participants are comfortable with each other. Others experienced that participants dropped out of the panels or focus groups. One representative mentioned that involving participants could be expensive if they were geographically widespread. Several cohort studies (*N* = 8) provided participants with a low value honorarium or a certificate of participation and reimburse travel costs. However, the representatives feared that using incentives may attract the wrong participants or be seen as suspicious.

### Resources and reflections on PEI

Resources to work on PEI largely varied between cohort studies. A few resourceful cohort studies had several members of staff working full-time with communication and engagement. However, most studies only had a part-time or full-time employee to work with PEI, and a few were able to increase their staff allocated to PEI during data collection waves.

The representatives explained that human resources are indispensable to raise awareness among participants about what research entails, to tailor information to the needs of different participant groups and maintain contact between data collection waves. They however experienced that PEI resources were insufficient and identified future needs. The representatives primarily hoped for more human, technical, educational, and financial resources. Communication resources may include staff to work in a targeted manner with different types of underrepresented cohort sub-groups such as young men, families, and single parents, and to tailor communication to their needs. The representatives also wanted permanent staff to raise the cohort profiles and awareness through e.g., science festivals, public talks, podcasts, videos, TV shows, social media, and infographics, also between data collection waves. Several representatives pinpointed the need to have staff that can work locally and approach participants, for instance, in primary care, schools and community centres. One representative hoped for hiring staff to establish and coordinate networks of participants who can attend workshops on a short notice. Another one mentioned the need to have employees who can engage with participating after working hours to adapt to people’s personal agendas.

The representatives hoped for the development of technical solutions such as portals providing participants with real-time information about the projects they participate in, the data collected and data uses, future visits, results from individual measurements and summary statistics. One representative wished for a system that would quickly identify and invite groups to recruit for sub-studies, for instance, young people living in rural communities, and would help re-identify participants lost to follow-up. The representatives also mentioned the need for resources to train researchers in PEI and improve the health literacy of participants and awareness of research processes.

The representatives believed that funding for PEI should be secured, and PEI conducted as a fully integrated component of any research. They emphasized that funders should be made aware of the amount of time and effort retaining participants requires. Finally, several representatives believed that collaboration between cohorts should be encouraged as cohorts often work in silos. Developing best practices for PEI may be useful to learn from the successes and failures from other cohorts and provide evidence about efficient PEI strategies.

## Discussion

Longitudinal cohort studies are central to understand factors influencing health and disease across generations. The cohort studies in our sample represent some of the most well-established, long-running studies that are contributing to a wealth of research findings. These studies applied a variety of strategies to *engage* and *involve* participants with the objective to maintain their interest, willingness to contribute, and long-term commitment. Whereas the cohort representatives experienced that some strategies were efficient to recruit participants, collect their data, provide them with information and interact with them, they often chose PEI strategies based on resources available and word of mouth rather than solid evidence regarding potential effectiveness and impact. The representatives emphasized that human, technical, educational and financial resources remain insufficient to fully address current needs and called for the development of best practices to inform future choices.

The cohort studies in our sample aimed to gradually move toward a digitalization of PEI, as observed in other studies [36, 37] and elsewhere in the health research sector [38, 39]. The cohort representatives had some experience using digital tools to recruit participants [40], design questionnaires [41], and collect biological samples [42]. They mentioned that the digitalization of PEI is necessary but brings new challenges that require consideration, and they wondered whether investing in tools such as apps, is worth the effort. Securing return on investment may be challenging, as digital platforms are expensive to develop and maintain and require expertise to be designed in line with stringent privacy and confidentiality requirements and in a user-friendly manner [43]. They also need to be met as relevant and up to date by participants to be used. The potential exclusion of participants without technical skills or access to digital infrastructure suggests a need for accompanying digital tools with offline approaches [44, 45]. Recent studies have shown that digital solutions may bring opportunities to alleviate administrative burdens, increase effectiveness [46], improve data quality, and facilitate the follow-up of participants migrating or living in geographically remote areas [47, 48]. Exploiting these opportunities and ensuring transparency while minimizing short and long-term risks to participants, will likely be of importance in the future. A recent review by Oakley-Girvan et al. also suggests that providing personalized content in digital platforms may help secure long-term use [49]. More evidence will however be needed regarding which digital tool designs are cost-efficient, secure, inclusive of diverse groups of participants, and sustainable [36].

Several cohort studies in our sample aimed to become multi-generational. While original recruitment phases targeted well-defined groups such as twins and pregnant women, new waves of recruitment will likely imply the inclusion of participants with varying profiles, ages, and personal circumstances. Finding good solutions to engage groups such as young people, immigrants, ethnic minorities, men, and the elderly, was a particular concern among our representatives. To engage young people, the authors of a 2019 scoping review proposed giving them the possibility to decide how much participation they wish to have according to their abilities, interests, skills and availability to participate [50]. Other studies have identified engagement strategies such as designing activities that create a sense of ownership [51], allying with parents and families who can be influencers [21], and providing financial incentives to younger participants, and including this cost in the study’s budget [24]. A recent systematic review by Singh et al. suggested that engaging ethnic groups such as South Asian and Chinese participants may be facilitated by providing financial incentives, having research staff familiar with their cultural and historical background, and explaining the benefits of the study to them [52]. Other studies have emphasized the importance of building relationships and providing flexibility, for instance in the choice of communication platforms, to engage older participants [53]. Overall, results from these recent studies resonate well with the experiences of our representatives and demonstrate the need to build a solid evidence-base regarding which PEI strategies are efficient for specific groups.

Finally, the cohort representatives in our sample called for more cross-cohort collaboration to exchange experiences and develop best practices for PEI. They appreciated participating in our workshop to review results from our interviews and explained that such arenas are important to build competence in PEI across cohort studies. Networks exist to support longitudinal cohort studies in their PEI effort such as the Cohort & Longitudinal Studies Enhancement Resources (CLOSER) [54] and the Avon Longitudinal Study of Parents and Children (ALPSAC) engagement group [55], which are primarily sharing best practice across British and some European cohort studies. It might be worth considering broadening these networks to include more studies. To develop best practices for PEI, mapping existing recommendations, handbooks [56] and guidelines and gathering evidence from the literature may be useful as well as engaging researchers and participants in the identification of the most effective strategies.

In this study, we did not investigate which impact the PEI strategies used by the cohort studies had on retention rates. A recent scoping review reported that using diverse PEI strategies and offering flexibility to participants as well as frequent communication may be most efficient to maintain high retention rates [57]. Tailoring the PEI strategies to the specific needs of different cohort subgroups may also be useful. For instance, parents in pregnancy and birth cohort studies could be offered various participation methods such as online surveys, phone interviews or home visits to accommodate their preferences and schedules [58]. Such tailoring is practiced in disease-specific cohorts recruiting patients to adapt to their health status and situation, for instance by offering medical assessment at the patient’s home, and has shown to have positive impact on retention rates [59]. Future research could be conducted to compare PEI strategies across types of cohort studies, for instance, cohorts recruiting healthy participants vs. disease-specific cohorts recruiting patients, and to investigate how PEI strategies are tailored to different groups of participants. In the future, it will be important to identify tools to evaluate the impact of PEI strategies [56, 60] and to develop best practices that are clear, provide enough flexibility to adapt to different contexts, and are easily accessible.

### Study limitations

This study has important limitations. First, it included a sample of cohort studies that are well established, resourceful, and likely not representative of all longitudinal cohort studies. The cohort studies also included various groups of participants in terms of age and background, and we did not discuss in detail with the cohort representatives which PEI strategies were most suited for which groups. Although the cohort representatives checked our summary reports, some information about PEI strategies used in the cohorts may have been omitted, may not be complete or influenced by the personal views of the representatives. We should therefore be careful not to overgeneralize our results, and rather interpret them as a first insight into how cohort representatives work with PEI, and which challenges and opportunities they identify.

## Conclusions

This study showed that PEI is an essential component in the ecosystem of longitudinal cohort studies. Despite the variety of strategies in use, important knowledge gaps exist regarding approaches best suited for different participant groups, and likely impact. This is unfortunate in contexts where resources to work with PEI are limited. Promoting the exchange of experiences in a structured way, and developing best practices for PEI, was strongly recommended by our representatives and becomes urgent to ensure cohort sustainability over the long-term. Research funders who invest significant amounts of money in building and maintaining longitudinal cohort studies should prepare for the future and devote more resources to PEI and the development of best practices to secure the sustainability of their investments and maximize the benefits of researcher-participant interaction [61–63].

## Electronic supplementary material

Below is the link to the electronic supplementary material.

Supplementary Material 1

## Data Availability

The datasets used and/or analysed during the current study are available from the corresponding author on reasonable request.

## Abbreviations

PEI: Participant engagement and involvement
