# Supplementary material for: Participant engagement and involvement in longitudinal cohort studies: qualitative insights from a selection of pregnancy and birth, twin, and family-based population cohort studies
Source: BMC Med Res Methodol. 2024 Dec 3;24:297. doi: 10.1186/s12874-024-02419-8 (PMC11613753; doi:10.1186/s12874-024-02419-8)
Supplement: Supplementary file 1 — Supplementary Material 1 [file 12874_2024_2419_MOESM1_ESM.docx]

**SUPPLEMENTARY FILE**

**Participant engagement strategies – Interview guide**

*This interview is to discuss engagement practices in your cohort study.*

***By engagement practices, we mean:***

- ***Activities to recruit and collect the consent of participants***
- ***Activities to inform participants about the research***
- ***Activities to interact with participants to collect data and involve them in cohort activities***
- ***Activities to maintain the participants’ interest and motivation over time and reduce attrition***

*I will provide examples of what we mean when discussing engagement activities.*

*This interview is recorded to help us structure our notes.*

*The recording will be destroyed after transcription of the interview in an anonymous form.*

*The interview will last for approx. one hour.*

*Any questions before we start?*

**RECRUITMENT AND CONSENT**

**Q1: Have you recently recruited new groups of participants (i.e. who have not been part of your cohort before), or are you planning to recruit new groups? E.g., to substudies**

**IF YES:**

- Which groups?
- How do you reach potential new participants? E.g. ads in social media, newspapers
- Did you apply any specific strategies to recruit participants from groups generally underrepresented, e.g. based on socio-economic status or ethnic background?
- How do new participants enroll the study? E.g. using an electronic form
- How do you collect the consent of new participants? E.g. electronically

**Q2: For your established cohort (e.g. participants who are already part of the cohort), has there been a need to update or renew consent? E.g. young participants becoming adults**

**IF YES:**

- How do you collect the renewed/updated consent? E.g. on paper, electronically
- Can the participants manage their consent preferences using a digital platform?
   **IF YES:**

  - What options do they have? E.g. use the platform to consent to new studies

  - Did you receive any feedback from participants on the use of the platform? E.g. easy to use

How can participants withdraw their consent and what are the consequences of withdrawal? E.g. samples and data are no longer used in further analyses

**INFORMATION TO PARTICIPANTS**

*Now I will ask about how general information is provided to participants. E.g. information about what happens in the study such as new general results or new study waves*

**Q3: How do you provide general information about the study to participants? E.g. web site**

| *Let the respondent speak first, then probe with the following if not mentioned:*   - *Emails* - *SMS with links to information* - *Newsletters* - *Social media* - *Public conferences and events (e.g. visit to labs, cultural events)* - *Infographics and videos* |
| --- |

How frequently do you provide general information to participants?

Do you use the same information channels/methods with all participants, or do you differentiate methods depending on participant groups? E.g. Facebook groups for young people

In your opinion, how efficient are the information channels/methods you use to inform participants? E.g. how many followers on social media (if used)

*Now I will ask about how individual information is provided to participants.*

**Q4: Do you provide any individual information back to participants?
E.g. results from analysis of biological samples, diagnostic findings, genetic research results, or personalized health guidance.**

**IF YES:**

- What information do you provide and how? E.g. Log in/account for participants, F2F meeting
- Can participants choose NOT to receive individual information?
- Did you receive any feedback from participants regarding their access to individual information? E.g. they find beneficial to receive individual information

**DATA COLLECTION PROCEDURES**

*Now I will ask about how data are collected from participants to answer study questions.*

**Q5. How do you collect data from participants? E.g. online questionnaires**

| *Let the respondent speak first, then probe with the following if not mentioned:*   - *Paper-based questionnaires* - *Mobile devices/wearables (e.g. fitbit)* - *In-person measurements/interviews* - *Cohort app on mobile phone* - *Data linkages (e.g. to electronic health records, welfare payment data, geographical data)* |
| --- |

Do you use the same methods to collect data from all participants, or do you differentiate methods depending on participant groups? E.g. young people, poor responders

Do you make use of reminders? If yes, how and how frequently?

Do you experience that sending reminders positively affects the response rate?

Do you follow-up non-responders to inquire why they do not participate?

Do you provide your participants with any incentives in connection with new data collections? E.g. gift card, lottery

**IF YES:**

- Do you differentiate incentives depending on participant group? E.g. young people
- How do you decide on the value of the incentives? E.g. follow ethical/research guidelines
- In your experience, does the use of incentives impact response rates?

**IF NO:**

- Did you discuss the use the incentives in the cohort?

**PARTICIPANT INVOLVEMENT**

*Now I will ask about activities conducted to involve participants in the life of the study.*

**Q6. Do you consult with participants to discuss some aspects of the study such as its design, objectives and research directions? E.g. focus groups to discuss research questions**

**(Think of consultations conducted during the last 2 years)**

| *Let the respondent speak first, then probe with the following if not mentioned:*   - *Individual interviews* - *Participant or community advisory groups/panels* - *Participant ambassadors* - *Workshops (F2F or digital)* - *Townhall meetings*   *For each activity mentioned:*   - *Ask about objectives* - *Ask about use of incentives* |
| --- |

How do you use the feedback received from participants?

Have you discussed internally the usefulness of consulting participants and if yes, what did you learn?

**RESOURCES**

**Q7. What dedicated resources does your cohort have to work with participant engagement? E.g. full-time staff position to work specifically with participant engagement**

**Q8. If you had unlimited resources and support to conduct participant engagement, what would you envision to do to engage your participants?**

**CONCLUSION**

**Q9. In your experience, what do you perceive as main barriers/limitations to participant engagement in your cohort?**

**Q10. What kind of information and knowledge will you need in the future to pursue participant engagement? E.g. best practices**

**Q11. Is there anything else we have not discussed that is important to take into consideration to inform successful participant engagement strategies?**

**THANK YOU FOR YOUR TIME!**

Next steps:

- We will send you a report summarizing our discussion for comments and approval
- Later, we will contact you once we have interviewed all cohorts and start with the data analysis
